# Distinct Signature of Altered Homeostasis in Aging Rod Photoreceptors: Implications for Retinal Diseases

**DOI:** 10.1371/journal.pone.0013885

**Published:** 2010-11-08

**Authors:** Sunil K. Parapuram, Radu I. Cojocaru, Jessica R. Chang, Ritu Khanna, Matthew Brooks, Mohammad Othman, Sepideh Zareparsi, Naheed W. Khan, Norimoto Gotoh, Tiziana Cogliati, Anand Swaroop

**Affiliations:** 1 Department of Ophthalmology and Visual Sciences, Kellogg Eye Center, University of Michigan, Ann Arbor, Michigan, United States of America; 2 Neurobiology Neurodegeneration & Repair Laboratory, National Eye Institute, National Institutes of Health, Bethesda, Maryland, United States of America; 3 Howard Hughes Medical Institute-National Institutes of Health Research Scholars Program, Bethesda, Maryland, United States of America; University of Florida, United States of America

## Abstract

**Background:**

Advanced age contributes to clinical manifestations of many retinopathies and represents a major risk factor for age-related macular degeneration, a leading cause of visual impairment and blindness in the elderly. Rod photoreceptors are especially vulnerable to genetic defects and changes in microenvironment, and are among the first neurons to die in normal aging and in many retinal degenerative diseases. The molecular mechanisms underlying rod photoreceptor vulnerability and potential biomarkers of the aging process in this highly specialized cell type are unknown.

**Methodology/Principal Findings:**

To discover aging-associated adaptations that may influence rod function, we have generated gene expression profiles of purified rod photoreceptors from mouse retina at young adult to early stages of aging (1.5, 5, and 12 month old mice). We identified 375 genes that showed differential expression in rods from 5 and 12 month old mouse retina compared to that of 1.5 month old retina. Quantitative RT-PCR experiments validated expression change for a majority of the 25 genes that were examined. Macroanalysis of differentially expressed genes using gene class testing and protein interaction networks revealed overrepresentation of cellular pathways that are potentially photoreceptor-specific (angiogenesis and lipid/retinoid metabolism), in addition to age-related pathways previously described in several tissue types (oxidative phosphorylation, stress and immune response).

**Conclusions/Significance:**

Our study suggests a progressive shift in cellular homeostasis that may underlie aging-associated functional decline in rod photoreceptors and contribute to a more permissive state for pathological processes involved in retinal diseases.

## Introduction

Aging is a complex biological process influenced by stochastic occurrence of molecular damage over time, but despite inherent randomness, the hallmarks of aging appear to be specific and uniform between individuals and even among species [1]. Genetic and expression profiling studies have led to the identification of conserved pathways that influence longevity, aging-associated phenotypes, and disease processes [2], [3]. Pioneering studies in aging have converged upon oxidative phosphorylation [4], [5], [6], insulin/insulin-like growth factor 1 signaling [7], [8], and caloric restriction signaling [9], [10], [11] as key cellular pathways, establishing a molecular basis for the fundamental connection between metabolism and longevity. A highly-conserved age-related change in neurons and other post-mitotic cells is decrease in expression of genes associated with oxidative phosphorylation, corresponding to a decline in mitochondrial function and an increase in reactive oxygen species (ROS) [1], [4]. Increased ROS and/or environmental insults can cause cellular damage, which triggers stress response pathways. Stress response constitutes another broad category of conserved age-related pathways that encompass a variety of biological processes, from immune regulation to epigenetic modification [12], [13], [14].

Despite a broad consensus, many changes in gene expression appear to be tissue- or even cell-type specific [1], [15], [16], [17], reflecting adaptive shifts in cellular homeostasis. For example, different tissues employ distinct mechanisms to respond to stress. Neurons may silence damaged DNA regions, while proliferating cells engage tumor suppressor genes [14], [18]. Response to exogenous antioxidants [19] and caloric restriction [20] also exhibits tissue-specificity. Hence, investigations of tissue- and cell-type specific age-related changes are critical to elucidate mechanisms of underlying pathophysiology and design treatments for tissue-specific aging-associated diseases.

Age is a major risk factor for age-related macular degeneration (AMD), a leading cause of untreatable vision loss in the elderly [21], [22], [23]. Advanced age also exacerbates the clinical outcome of genetic defects in other retinopathies [24], yet the contribution of aging to retinal disease(s) remains largely unspecified. A few studies have examined age-related gene expression changes in the retina of humans and mice and suggested aging-associated alterations in genes involved in energy metabolism, stress response [25], lipid metabolism [26], and transcriptional regulation [27]. However, these reports have limitations with respect to time points, number of genes, and heterogeneity of samples studied. Additionally, averaging gene expression changes of the entire retina may obscure subtle yet important age-related alterations in individual cell-types.

Rod photoreceptors represent the predominant retinal cell type in most mammals and are uniquely vulnerable to the effects of age. In the human retina, about 30% of central rods are lost by the ninth decade, whereas cone density remains essentially unchanged [28], [29]. There is a corresponding decline in rod-mediated visual function with increasing age [30]. A similar pattern of rod vulnerability to age-related functional decline is observed in mice [31]. More significantly, in retinitis pigmentosa and AMD, the loss of rods precedes cone loss [32], [33], [34]. Notably, rods also produce trophic factors for cone survival [35], [36]. Therefore, characterizing the adaptive response of rod photoreceptors to increasing age should be valuable for delineating the biology of retinal aging.

We had previously developed mice that express enhanced Green Fluorescent Protein (eGFP) under the control of the Neural Retina Leucine zipper promoter (Nrlp), which restricts eGFP expression in the retina to developing and mature rod photoreceptors [37]. In this study, we have taken advantage of the Nrlp-eGFP mice to identify candidate genes and pathways underlying rod photoreceptor vulnerability to aging. We hypothesized that relevant adaptive changes in gene expression may occur well before pathological manifestations of aging, such as the reduction in the outer nuclear layer (ONL) thickness and/or alterations in electroretinogram (ERG) recording [38], become apparent. We have compared gene expression in purified populations of mouse rod photoreceptors using the age of reproductive maturity (1.5 months) as a baseline. To capture early age-related changes, we chose adult time points of 5 months and 12 months, representing early aged states with little measurable functional decline in the mouse retina, and that correspond to the late twenties and early forties, respectively, in humans (http://research.jax.org/faculty/harrison/ger1vLifespan1.html and [39]). Our results show that changes in metabolic and signaling pathways, including the consensus pathways implicated in aging, are evident as early as 5 months of age. Our study discusses a new perspective on the potential interplay between normal aging and age-related retinal diseases.

## Results

### Nrlp-eGFP mice show normal changes in retinal histology and function with age

**Figure 1:**
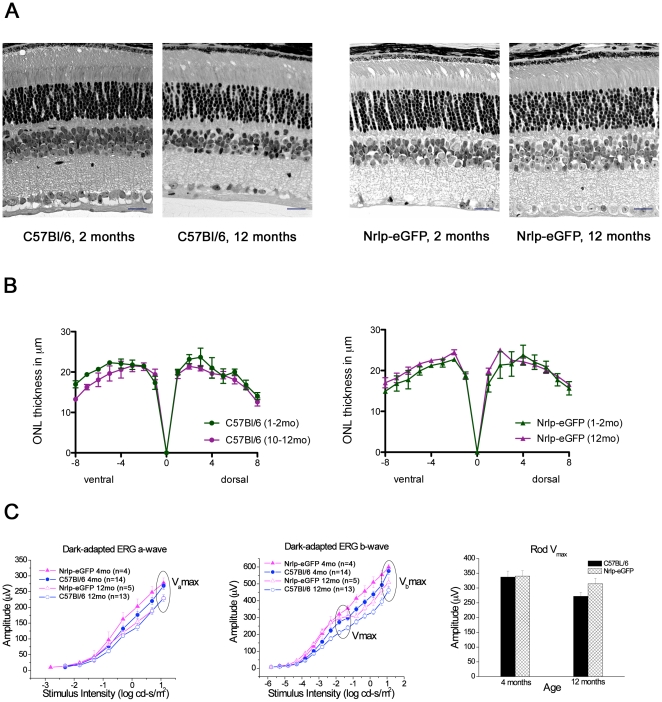
Comparison of young and old C57Bl/6 and Nrlp-eGFP retinas. (A) Representative 10 µm plastic sections of central retina from 2 and 12 month old C57Bl/6 and Nrlp-eGFP mice show a 10–12 nuclei thick outer nuclear layer (ONL) and no obvious changes in morphology of inner or outer segments. Scale bar, 20 µm for all microphotographs. (B) ONL thickness measured in a representative section taken at the optic nerve head was also unchanged in both mouse strains at the two ages (n = 3 for each). (C) Dark-adapted ERG a- and b-wave and rod V_max_ for Nrlp-eGFP and C57Bl/6 mice at 4 and 12 months of age.

We first evaluated whether eGFP expression in rod photoreceptors had any effect on the physiology or cellular morphology by comparing Nrlp-eGFP mice to wild-type (C57Bl/6) mice at different ages. Nrlp-eGFP mice developed normally and showed no histological signs of retinal degeneration by 12 months of age (Figure 1A). ONL thickness was comparable in Nrlp-eGFP and C57Bl/6 mice and remained unchanged by 12 months of age in both strains (Figure 1B). Dark-adapted ERG was characterized by rod-mediated scotopic maximum amplitude (V_max_), sensitivity *k*, and a- (Va_max_) and b-wave (Vb_max_) amplitudes at maximum flash intensity of 1.09 log cd-s/m^2^. ERGs of Nrlp-eGFP mice showed a mild decline with age in rod function similar to that of age-matched C57Bl/6 controls (Figure 1C). There was no appreciable difference between young and old mice in dark-adapted, rod-mediated a- and b-wave thresholds at 25 µV; however, the amplitude of the response diverged and became more pronounced as the flash intensity increased. At maximum flash intensity, the dark-adapted a-wave, Va_max_, which reflects rod photoreceptor response to light, was significantly reduced in Nrlp-eGFP and C57Bl/6 mice at 12 months compared to 4 month old mice (p<0.02 and p<0.04, respectively). The dark-adapted b-wave, Vb_max_, which reflects signaling downstream of the photoreceptors, declined with age in Nrlp-eGFP and C57Bl/6 mice (p<0.03 and p<0.001, respectively). Rod-mediated scotopic amplitude V_max_, also declined with age but was not significantly different in Nrlp-eGFP as it was the case for C57Bl/6 mice (p<0.01). There was no change in sensitivity *k* with increased age in either group. For all ERG parameters, age-dependent changes in Nrlp-eGFP mice mirrored those observed in C57Bl/6 mice. Expression of eGFP in rod photoreceptors, therefore, does not appear to cause any significant difference compared to C57Bl/6 mice in retinal histology or function up to 12 months of age, though a slight decline in rod function by 12 months of age is detected in both strains.

### Microarray analysis identifies a signature gene expression profile of aging rod photoreceptors

**Figure 2:**
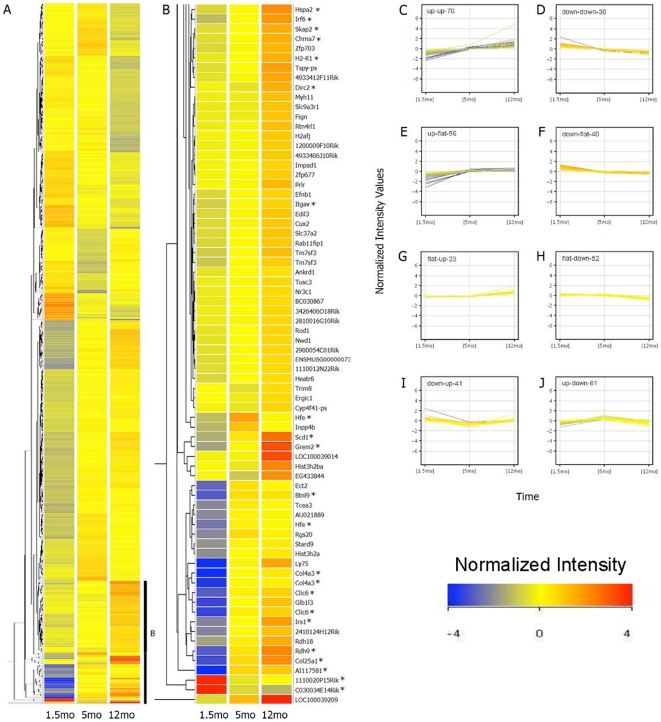
Hierarchical clustering and profile plot view. Four biological replicates were used for microarray hybridization on GeneChip Mouse Genome 430 2.0 Arrays. (A) Hierarchical clustering dendrogram of 413 probes that have a False Discovery Rate (FDR) p≤0.05 and a minimum fold change 1.5 between at least two of the three time points (1.5, 5, and 12 months). Bright blue indicates lowest signal with increasing values indicated by yellow shading to bright red, representing peak signal. (B) Magnified view of genes with high fold-change that exhibit increasing expression with age. * indicates evaluated by qPCR. (C–J) Profile plot view corresponding to the 413 entities. Normalized expression values are shown on the *y* axis and the three time points are shown on the *x* axis. The number of genes in each cluster is indicated in the top left corner of each graph. mo, month old.

To evaluate global gene expression changes, we purified rods from Nrlp-eGFP mice at 1.5, 5, and 12 months of age using flow cytometry, as described previously [37]. As the connecting cilium is fragile, rod outer segments are rarely retained after tissue dissociation, even prior to sorting. However, eGFP is maintained in the cell body of dissociated rods, and the fluorescence allows selective sorting of eGFP-positive cells. After sorting, 98% of the cells are alive and, upon re-sorting, 100% of these are eGFP-positive (data not shown). No significant difference was detected in the average number of purified cells obtained from a single animal between 1.5 and 12 months (1.04×10^6^ and 9.54×10^5^, respectively; n = 4, p = 0.846 by Student's two-tailed t test). Gene expression was compared among the three selected time points by one-way ANOVA that yielded 413 probe sets (corresponding to 375 transcribed sequences) showing >1.5-fold change at p<0.05 between at least two of the three time points (Figure 2A, B and Table S1). Eight basic patterns of expression are depicted in Figure 2C–J. A majority of the probe sets (58%) showed an altered expression at 5 months of age relative to 1.5 months, with continued change in the same direction at 12 months.

### Validation and specificity of age-related gene expression changes in rod photoreceptors

**Figure 3:**
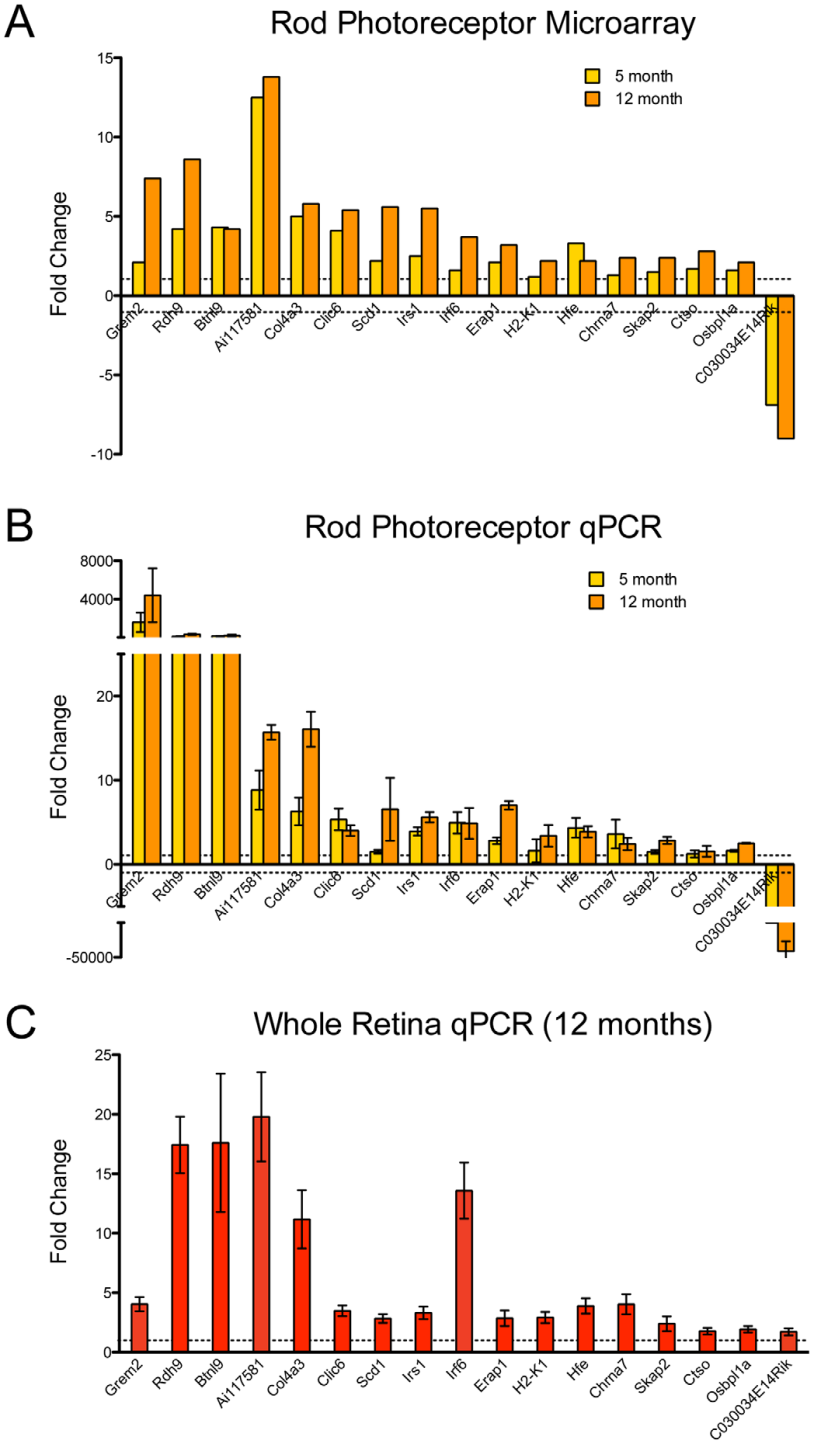
qRT-PCR validation of selected genes using independent biological samples. Predicted fold changes from microarray analysis and relative gene expression fold change from qRT-PCR (y-axis, expression normalized relative to 1.5 month old group, represented by dotted lines at y = 1, −1) for independent biological replicates of rod photoreceptors (n = 4 for each experiment) is shown in (A) and (B), respectively. The same genes were tested on whole retina samples from Nrlp-eGFP mice aged 1.5 and 12 months, n = 4 biological replicates for each time point (C). Error bars indicate ±SEM. *Grem2* expression was detected only beginning at 5 months in photoreceptors.

Twenty-five genes with at least 2-fold change and/or potential biological relevance were selected for validation by quantitative RT-PCR (qRT-PCR) using independent rod photoreceptor RNA samples (n = 4 biological replicates). Seventeen of the 25 genes exhibited changes in the predicted direction at both 5 months and 12 months of age relative to 1.5 months; of these, 16 showed increased expression with age, and one showed reduced expression (compare Figure 3A and B). Of the remainder, four genes demonstrated changes in expression in the predicted direction for either 5 or 12 months relative to 1.5 months, whereas the remaining four did not change in the direction predicted by the microarray analysis (Table S2). The list of validated genes includes those associated with retinoic acid receptor (RAR) signaling - retinol dehydrogenase 9 (*Rdh9*); immune system regulation - histocompatibility 2 K1, K region (*H2-K1*), interferon regulatory factor 6 (*Irf6*), and endoplasmic reticulum aminopeptidase 1 (*Erap1*); lipid and insulin metabolism - stearoyl coenzyme A desaturase (*Scd1*), oxysterol binding protein-like 1A (*Osbpl1a*), and insulin receptor substrate 1 (*Irs1*); and two genes that have not yet been characterized (*Ai117581, Riken C030034E14*).

To evaluate whether gene expression changes in our analysis were characteristic of rod photoreceptors, we performed qRT-PCR analysis of the 17 validated genes using total RNA from whole retina from young (1.5 month old) and old (12 month old) Nrlp-eGFP mice (Figure 3C). Whole retina RNA showed significant age-related changes that were consistent in direction compared to isolated photoreceptors for 16 out of 17 genes (Figure 3C). The magnitude of fold change, however, was at least tenfold higher in photoreceptor samples than retina samples for *Grem2*, *Rdh9*, and *Btnl9*. *Irf6* demonstrated a higher magnitude of change (over twofold) in retina samples than photoreceptor samples, but the remaining genes exhibited comparable fold changes with age. Expression of only one gene, C030034E14Rik, changed in an opposite direction with age in whole retinas compared to isolated rod photoreceptors.

### *In silico* analyses converge on pathways and processes associated with photoreceptor aging

Computational approaches were adopted to identify overrepresented biological pathways among our list of 375 age-related differentially expressed genes. Multi-functional proteins were accounted for in each pathway/category to which they belong. First, we used gene ontology (GO) enrichment analysis [40] to assess overrepresentation of specific GO terms. We chose the broadest categories within “Cellular Process” with an enrichment score >3 [40] that contained at least 2 genes from our list. Twenty-one GO terms met these set criteria, with *Eye Morphogenesis*, *Electron transport chain*, *Motor Axon Guidance*, and *Antigen presentation* among the top ten (Table 1). Some of the altered cellular processes might reflect remodeling and adaptation during rod photoreceptor aging.

**Table 1:** Gene ontology (GO) enrichment analysis of aging-associated genes in rod photoreceptors.

*Cellular Process*	*Ratio* 1	*Score* 2
Eye Morphogenesis	0.182	8.30
Nucleosome Assembly	0.059	8.05
Electron Transport Chain	0.067	7.76
Motor Axon Guidance	0.143	6.59
Determination of Bilateral Symmetry	0.086	5.30
Response to nutrient level	0.111	5.09
Regulation of protein localization	0.079	4.79
Sperm motility	0.200	4.55
Antigen presentation via MHCI	0.095	4.30
Regulation of DNA metabolism	0.091	4.09
Cell cycle arrest	0.065	3.73
Transcription	0.026	3.72
Intracellular signaling cascade	0.031	3.62
Phagocytosis	0.080	3.52
Angiogenesis	0.048	3.51
Cell morphogenesis	0.040	3.19
Anatomic structure formation	0.040	3.16
Regulation of transmission for nerve impulse	0.058	3.14
Transmembrane transport	0.045	3.08
Chromosome segregation	0.071	3.07
Regulation of transcription	0.025	3.02

1 Number of genes from our dataset divided by the total number of genes that are present in the given cellular process category.

2 Log transformation of the p-value calculated using a chi-square test comparing the proportion of our gene list in a given GO group to the proportion in the background (i.e., not in the given GO group).

Ingenuity® Pathway Analysis (IPA) sorts genes into canonical pathways based on the scientific literature and indicates which are significantly overrepresented [41]. Eight canonical pathways with p≤0.01 are listed in Table 2. Our microarray data showed reduced expression with age for genes encoding proteins in complex I, III, and V of the oxidative phosphorylation pathway, whereas expression of genes in RAR activation pathway (such as *Rdh9*) mostly increased. Both higher and lower expression changes were detected with age for genes in the tight-junction signaling and Cxcr4 signaling pathways.

**Table 2:** Canonical pathway analysis of aging-associated genes in rod photoreceptors.

*Canonical Pathways*	*Age-Related Genes*	*Ratio* 1	*P-value* 2
Oxidative Phosphorylation	*Ucrc, Atp5d, Ppa2, Uqcr, Uhrf1bp1, Ndufa3, Uqcrb, Atp6v1b2, Ndufa13*	0.0542	0.001
Tight Junction Signaling	*Mylk, Epb41, Fos, Rhoa, Jam2, Prkaca, Akt3, Myh11*	0.0488	0.003
Glucocorticoid Receptor Signaling	*Taf4b, Fos, Ikbkg, Pou2f1, Chp, Prkaca, Akt3, Nfatc2, Ncor1, Hspa2, Nr3c1*	0.0393	0.004
CXCR4 Signaling	*Fos, Gng11, Itpr2, Cxcl12, Rhoa, Akt3, Pak7, Prkd1*	0.0476	0.005
PXR/RXR Activation	*Ppara, Scd, Prkaca, Akt3, Nr3c1*	0.0581	0.007
RAR Activation	*Rdh9, Fos, Rarb, Prkaca, Akt3, Ncor1, Smad5, Prkd1*	0.0449	0.007
Prolactin Signaling	*Fos, Irs1, Prlr, Nr3c1, Prkd1*	0.0641	0.008
Aryl Hydrocarbon Receptor Signaling	*Ahrr, Gsta3, Ccna2, Fos, Aldh1l2, Rarb, Cdkn1b*	0.0446	0.010

1 Number of genes from the data set in the given pathway divided by the total number of genes that map to that given canonical pathway.

2 Derived using Fisher's exact test to determine the probability that the association between the 413 probesets in our dataset and the canonical pathway in the Ingenuity Pathways Knowledge Base is explained by chance alone.

Ingenuity® network analysis revealed biological processes that were enriched among the 100 genes showing progressively higher or lower expression across the three time points (see Figure 2C–D). We then selected biological processes associated with a minimum of four genes in the resulting networks. We observed a clustering of genes for angiogenesis, apoptosis, and transcription (Table 3). Genes involved in regulation of transcription exhibited decreased expression with age. The genes associated with angiogenesis and apoptosis revealed variable expression changes, but those showing increased expression with age tend to promote angiogenesis and oppose apoptosis.

**Table 3:** Key biological processes associated with rod photoreceptor genes showing progressive change with age.

*Biological Process*	*Expression Change* 1	*Genes*
Angiogenesis	↑	*Chrna7, Edil3, CD59a, Hpse*
	↓	*Acvrl1, Cxcl12*
Transcription	↓	*Dnmt3a, Tef, Neurl, Ncor1*
Apoptosis	↑	*Akt3, Cast*
	↓	*Cxcl12, Rhoa*

1 Refers to expression change at 5 and 12 months, compared to 1.5 months.

“↑” indicates increased expression relative to 1.5 months,

“↓” indicates decreased expression.

**Figure 4:**
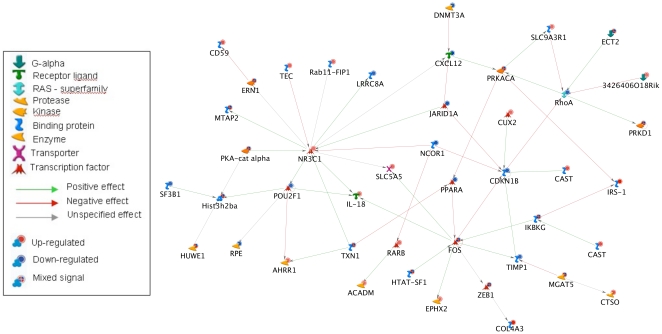
Network Analysis. Direct interactions among the 375 age-related genes. Arrow color indicates the type of interaction, symbol shape indicates the type of gene product/protein, and background symbol color indicates direction of change in expression with age, blue indicating decreasing expression and red indicating increasing expression with age. This network was generated using MetaCore™ from GeneGo, Inc. (www.genego.com).

Finally, we generated a network reconstruction using MetaCore™ from GeneGo, Inc. [42] to explore what direct interactions may exist among the entire set of 375 differentially expressed genes (Figure 4). The following gene products emerged as hubs: Fos, a transcription factor involved in stress response; glucocorticoid receptor (encoded by *Nr3c1*), involved in stress response and inflammation; RhoA, an important signaling molecule associated with cytoskeletal remodeling; and Cdkn1b, which maintains cell cycle arrest. Additionally, several genes involved in overrepresented pathways (described above) were present in the direct interaction network. Overall, genes associated with stress response appeared to occupy prominent positions (hubs) in the network topology.

## Discussion

Aging-associated changes occur on multiple levels and contribute to cellular dysfunction and disease. Landmark studies in yeast and *C. elegans* have identified a genetic basis for the modulation of aging phenotypes that extends to higher organisms [2], [9]. Similarly, genome-wide expression profiling studies have revealed candidate genes that reinforce consensus pathways but also demonstrate tissue-specificity [1], [13], [16], which likely has implications for aging-associated diseases of specific tissues. The retina provides an ideal paradigm to dissect molecular events associated with neuronal aging *in vivo* as it is the most accessible part of the central nervous system. Furthermore, evidence suggests shared characteristics between age-related diseases of the retina and the brain, such as AMD and Alzheimer's disease [23]. In particular, neuronal degeneration in both diseases is closely associated with vascular damage. Neurodegeneration in the retina (and more specifically, degeneration of photoreceptors) is observed in numerous syndromic diseases (www.sph.uth.tmc.edu/Retnet/) and is uniquely amenable to non-invasive evaluation, which facilitates the study of natural history and potential therapies. However, the direct impact of aging on molecular pathways that guide photoreceptor metabolism or function has yet to be elucidated. Here, we report adaptive changes in both conserved and cell-specific age-related pathways through temporal profiling of purified rod photoreceptors. We show that a majority of these expression changes begin in young adults and continue to progress with age. Furthermore, our study reveals that single cell-type profiling provides better resolution of subtle and progressive age-related expression changes (see below), yielding a more comprehensive depiction of cellular aging.

**Figure 5:**
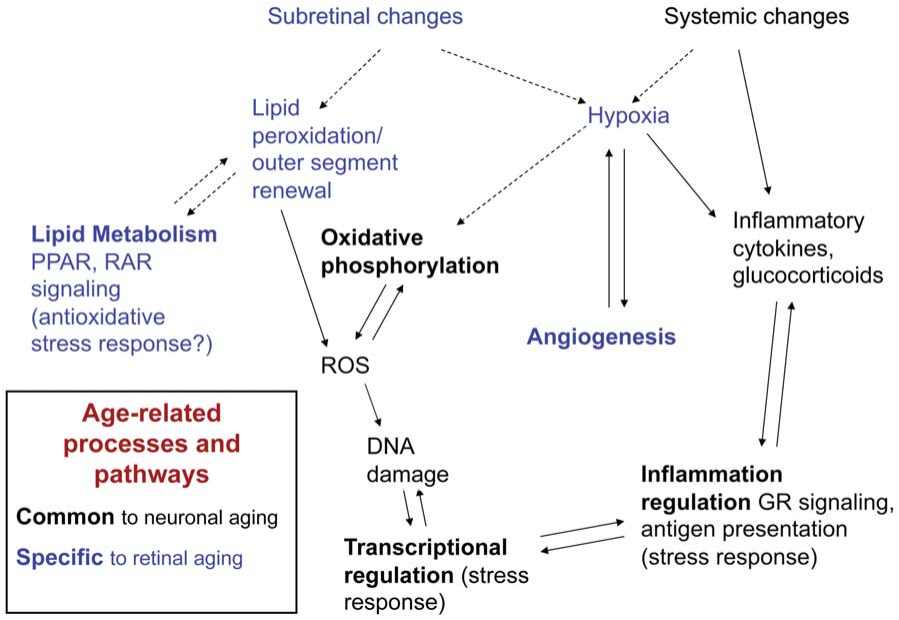
Diagram of hypothesized age-related pathways relevant to rod photoreceptors. Environmental and intracellular stressors such as hypoxia and reactive oxygen species (ROS) induce gene expression changes in consensus pathways of aging (transcriptional regulation, oxidative phosphorylation, and inflammation, labeled in black), as well as pathways specific to retina and photoreceptors (angiogenesis and lipid metabolism, labeled in blue). ROS, reactive oxygen species; GR, glucocorticoid receptor.

Though rod photoreceptors are highly specialized neurons involved in photoreception, we identified changes in consensus pathways of aging, including oxidative phosphorylation and stress response affecting transcription and inflammation (Figure 5). Rods are highly metabolically-active post-mitotic neurons that are exposed to oxidative stress [43], and a decline in electron transport chain (ETC) efficiency is thought to create increased ROS that can further damage DNA, lipids, and other molecules [3], [5]. Photoreceptors rely on oxidative phosphorylation, unlike most cell-types of the retina [44]. Expression of ETC genes is shown to decrease with age in many tissues and species [1], [4]. Reduced expression of ETC genes is evident in profiles of aging rods, reported here, but this pathway was not identified in previous profiling studies of whole retinas [26], [27]. Furthermore, some of the expression changes we observed in rod photoreceptors were quantitatively different (Grem2, Ai117581 and Irf6) or in the opposite direction (C030034E14Rik) in 12-month old whole retina. Further investigations of this novel gene may provide insights into the differences in pattern of expression in photoreceptors compared to whole retina. Thus, our findings provide an example of the advantage of investigating specific cell-types in addition to the whole tissue.

Patterns of histone acetylation and DNA methylation have been shown to change with age (and caloric restriction) from yeast to humans, influencing DNA stability and gene expression patterns [45], [46]. Our data show a significant enrichment of epigenetic processes including *regulation of transcription* and *nucleosome assembly*, and expression of genes for histone clusters 1, 2, and 3 (*Hist1h3b, H2afj, Hist3h2a, Hist3h2ba*) progressively increased over time (Table S1). DNA-modifying genes that changed with age include DNA methyl-transferase 3 alpha (*Dnmt3a*) and Suppressor of variegation 4–20 homolog 1 (*SUV420h1*), which was also noted to change with age in human retinas [27]. Additionally, transcriptional repressors Hairless (*Hr*) and Nuclear co-repressor 1 (*Ncor1*) both decreased with age, suggesting a derepression of transcription that has been associated with epigenetic changes in other aging cells [47]. Chronic transcriptional changes may therefore contribute to increasing susceptibility to damage with age, causing dysfunction and ultimately cell death.

Upregulation of inflammatory response pathways has been associated with neuronal aging of rodents and humans [48]. Like brain parenchyma, the neuroretina exists in an immune-privileged state, but this state is compromised in age-related retinal diseases such as AMD and diabetic retinopathy [49]. Increases in interferon-regulated transcription factor *Irf6* and antigen presentation genes *Erap1* [50] and *H2-K1* suggest a similar upregulation of inflammation signals in photoreceptors. Increased expression of *H2-K1* and the human homolog, *HLA-C*, was also reported in previous studies of aging mouse and human retina and eye tissue [1], [26], [27]. Pathway and network analyses also suggest potential inflammation-modifying changes in *Fos* and *Nr3c1* (glucocorticoid receptor) signaling that may contribute to systemic and cognitive aging [3], [51], [52], [53]. We propose that stress response may induce a transition of photoreceptors to a state more likely to trigger immune system activation as well as a potential decline in immune-privilege, which may contribute to pathology and neurodegeneration.

Stress response processes, including angiogenesis and retinoid/lipid metabolism, are especially relevant for the aging retina (Figure 5). Photoreceptor function is intimately associated with retinal pigment epithelium (RPE) and choroidal blood supply [54]. Age-related changes in the neural retina microenvironment (such as ischemia, hypoxia, and inflammation) contribute to angiogenic signaling, and angiogenesis is a key feature of age-related retinal diseases [30]. Our findings indicate that a shift towards pro-angiogenic signaling may also be a feature of photoreceptor aging. Of the genes that progressively increased with age, *CD59a* is particularly interesting as it protects against complement attack [55], which plays a central role in AMD pathophysiology [56].

Changes in retinoid and lipid metabolism via the retinoic acid receptor (RAR) pathway and the peroxisome proliferator-activated receptor alpha (PPAR-alpha, encoded by *Ppara*) appear to play a role in photoreceptor aging. Altered retinoic acid metabolism has been implicated in some forms of retinal degeneration [57], [58] and in the regulation of angiogenic signals [59]. Expression of retinol dehydrogenases 9 and 18 (*Rdh9* and *Rdh18*) increased dramatically with age in rod photoreceptors, perhaps representing a stress response to lipid peroxidation, another source of ROS [60], [61]. Rod outer segment membranes have a uniquely high concentration of specific long-chain polyunsaturated fatty acids (LCPUFAs) that play an important role in phototransduction [62] and are also highly susceptible to peroxidation [63]. Extensive evidence points to the role of lipid peroxidation by-products in Bruch's membrane in inducing choroidal neovascularization [64], [65], [66], [67], [68]. We also observed age-related increases in *Scd1* and *Osbpl1a*, and a progressive decrease in *Ppara*, all of which influence lipid and insulin signaling [69], [70], [71]. Decreased expression of *Ppara* has also been observed in aging heart, liver, and kidney [72] and recently it has been broadly implicated in aging and systemic inflammation [73]. In rod photoreceptors, altered expression of *Ppara* may represent a response to age-related changes in RA signaling or lipid peroxidation, since LCPUFAs and retinoids are endogenous ligands for PPAR-alpha [74]. Our data suggest that intrinsic changes in such pathways may be important in the normal aging of rod photoreceptors.

Another aspect of our profiling and pathway analysis requires further discussion. Unlike exon arrays, 3′ microarray data cannot be considered as strictly quantitative. Gene lists are selected by statistical methods based on highest probability and although we corrected for false positives, the latter could not be completely excluded. Thus, qPCR validation is necessary for follow-up experiments as it provides higher sensitivity and reliability at the single gene level. Of the 25 genes that were verified by qPCR, 70% showed changes of expression concordant with microarray at both time points analyzed, with an additional 20% being validated for expression changes only at one time point. Furthermore, some of the expression changes, reported here, have been confirmed in our more recent exon array experiments (unpublished observations), and further investigations are in progress to examine their physiological relevance in rod photoreceptor aging. In addition to examining individual genes, we adopted a systems biology approach to analyze the expression data as a whole (macroanalysis). This type of analysis keeps the focus on groups of genes and the pathways/categories/functional groups they belong to, and it is best applied to larger unbiased data sets. The strength of the observation derives from the genes in similar pathways that behave in a coordinated fashion.

Our study provides an aging-associated gene expression signature for a single neuronal cell type. Studying rod photoreceptors instead of the entire retina has allowed us to identify significant age-related changes with better resolution. Both consensus and photoreceptor-specific age-related pathways, identified here, have implications for understanding the pathogenesis of AMD and other retinopathies. Notably, many expression changes are evident even at a relatively young adult age, suggesting that aging is a continuous process set in motion long before phenotypic changes are detected. Our studies reveal contributions of rod photoreceptors to retinal aging as well as potential changes in the immune-privileged status of the neuroretina with age. We cannot determine whether expression changes associated with normal aging are causative, permissive, or protective with respect to aging-associated functional decline and disease. Nonetheless, we propose that changes in gene expression represent an adaptive response of rod photoreceptors to microenvironment (such as exposure to light and oxidative stress) during the aging process. Identification of aging-associated pathways thus provides a foundation for future investigations and offers potential targets to develop treatments and therapies for age-related diseases.

## Materials and Methods

### Mice

All mice were used in accordance with the approved Institutional Animal Care and Use Committee (IACUC) protocol.

### Electroretinography

To evaluate retinal function, full field ERGs were recorded in Nrlp-eGFP mice and C57Bl/6 control mice at 4 and 12 months of age. Mice were dark-adapted overnight and were prepared under dim red illumination as described previously [75] after anesthesia with Ketamine (93 mg/Kg) and Xylazine (8 mg/Kg). Pupils were dilated with topical 1% Atropine and 0.5% Tropicamide. Body temperature was maintained at 37°C with a heating pad. Corneal ERGs were recorded from both eyes using gold wire loops with 0.5% tetracaine topical anesthesia and a drop of 2% methylcellulose for corneal hydration. A gold wire loop placed in the mouth was used as reference, and ground electrode was on the tail.

ERGs were recorded with a Ganzfeld configuration using the Espion e^2^ recording system (Diagnosys, MA) to brief xenon white flashes from −5.8 to +1.09 log cd-s/m^2^/flash in steps of 0.5 log units for dark-adapted responses and from −0.91 to +1.09 log cd-s/m^2^/flash over a 2 log unit range in steps of 0.3 log units for light-adapted responses. Responses were amplified at 1,000 gain at 1.25 to 1000 Hz, and digitized at a rate of 2000 Hz. A notch filter was used to remove 60 Hz line noise. Dark-adapted b-wave amplitudes were fitted with a Naka-Rushton function to determine estimates for the b-wave saturated amplitude,V_max_ and the response sensitivity, k. Light-adapted responses were recorded after 10 minutes of adaptation to a white 32 cd/m^2^ rod-suppressing background.

### Quantification of ONL thickness

Retinas from 1–2 and 10–12 months old Nrlp-eGFP and C57Bl/6 mice (n = 3 each) were dissected, fixed and processed to generate sagittal thin plastic sections encompassing the optic nerve head. The optic nerve was oriented in the section to indicate the ventral portion. ONL thickness was measured using Zeiss, AxioVision Documentation Rel.4.7 software on 40x-magnified images. Eight microscopy frames were counted ventrally and dorsally to the optic nerve head and measurements were taken in the middle of the frame. Values represent mean ± SEM.

### Isolation of rod photoreceptors

Rod photoreceptors were purified by flow-sorting, as previously described [37]. For qRT-PCR work, some samples were dissociated with dispase I (Roche Diagnostics Corporation, Indianapolis, IN) at 2units/mL for 45 min at 37C followed by trituration. Viscosity was reduced with DNase1 (10 mg/ml; 5 min at 37°C; Sigma-Aldrich). The eGFP-positive rod cells were flow-sorted from other dissociated retinal cells. RNA was extracted using the RNeasy Mini kit (Qiagen, Valencia, CA) and quality checked using the Agilent 2100 BioAnalyzer (Agilent Technologies, Inc., Santa Clara, CA).

### Target preparation for expression arrays

Five nanograms of total RNA from flow-sorted rod photoreceptors from Nrlp-eGFP mice at 1.5 month, 5 months and 1 year of age (n = 4 biological replicates for each time point) were used for target generation. Ovation RNA Amplification System V2 (NuGEN Technologies, San Carlos, CA) was used for cDNA amplification according to the manufacturer's protocol. FL-Ovation cDNA Biotin Module V2 (NuGEN Technologies) was used to fragment and biotin label the amplified cDNA and for hybridization of the labeled target onto a GeneChip Mouse Genome 430 2.0 Array (Affymetrix, Santa Clara, CA) with 45,000 probe sets for 34,000 well-characterized mouse genes. Washing and staining of the GeneChips was performed using the EukGE-WS2v4_450 protocol on the GeneChip Fluidic Station 450 (Affymetrix). GeneChip scanning was performed on the GCS3000-7G Scanner (Affymetrix).

### Microarray data analysis

The Robust Multichip Average (RMA) method [76] was used for background correction, quantile normalization, and to summarize the expression scores of the gene expression data. A one-way ANOVA was used to compare the three time points. To correct for false positives we used the Benjamini- Hochberg False Discovery Rate Method with a specified p-value cutoff of 0.05. This led to a set of 839 probes that have annotation information and then this set was subjected to a minimum fold change criteria of 1.5 between any of the three possible comparisons: 5 month vs. 1.5 months, 12 months vs. 1.5 months and 12 months vs. 5 months. The resulting set containing 413 probes was used for hierarchical clustering, canonical pathway, and network analysis using Ingenuity Pathway Analysis (Ingenuity® Systems, www.ingenuity.com, Redwood City, CA), MetaCore(TM) from GeneGO (GeneGo, Inc., www.genego.com, St. Joseph, MI), and GO Enrichment Analysis [40]. All data is MIAME compliant. Raw data has been deposited in the GEO database with accession number GSE22317.

### Quantitative RT-PCR

Retinas were dissected from four independent biological replicates for each of the three timepoints (1.5, 5, and 12 months), enzymatically dissociated as described above, and rod photoreceptors were flow-sorted into RNAprotect (Qiagen). Whole retinas were harvested from additional mice and snap-frozen. RNA was extracted using the RNeasy Mini kit (Qiagen) and quality was evaluated as described above. Amplified photoreceptor cDNA was derived using WT-Ovation Pico System (NuGEN), and whole retina cDNA was derived using Superscript II RT-PCR system (Invitrogen). cDNA was used for real-time qRT-PCR using SYBR(R) Green PCR Mastermix (Applied Biosystems, Foster City, CA) on the 7900HT Fast Real-Time PCR System (Applied Biosystems). Candidate genes were selected for analysis based on microarray predictions of fold-change >2, pattern of change, and gene function. Hypoxanthine guanine phosphoribosyl transferase (*Hprt*) and ribosomal protein S26 (*S26*) served as internal controls. Primer pairs tested are listed in Table S2.

## Supporting Information

Table S1 413 probe sets showed significant differential expression with age.
(0.24 MB XLS)

Table S2 25 genes evaluated by quantitative RT-PCR (photoreceptor data).
(0.02 MB XLS)
